# Identification of Early Heat and Water Stress in Strawberry Plants Using Chlorophyll-Fluorescence Indices Extracted via Hyperspectral Images

**DOI:** 10.3390/s22228706

**Published:** 2022-11-11

**Authors:** Mangalraj Poobalasubramanian, Eun-Sung Park, Mohammad Akbar Faqeerzada, Taehyun Kim, Moon Sung Kim, Insuck Baek, Byoung-Kwan Cho

**Affiliations:** 1 Department of Biosystems Machinery Engineering, Chungnam National University, Daejeon 34134, Korea; 2 Department of Smart Agricultural Systems, College of Agricultural and Life Science, Chungnam National University, Daejeon 34134, Korea; 3 Department of Agriculture Engineering, National Institute of Agricultural Science, Rural Development Administration, Jeonju 54875, Korea; 4 Environmental Microbial and Food Safety Laboratory, Agricultural Research Service, United States Department of Agriculture, Powder Mill Road, BARC-East, Bldg 303, Beltsville, MD 20705, USA

**Keywords:** strawberry, abiotic stress, chlorophyll-fluorescence indices, hyperspectral image, machine learning

## Abstract

Strawberry (Fragaria × ananassa Duch) plants are vulnerable to climatic change. The strawberry plants suffer from heat and water stress eventually, and the effects are reflected in the development and yields. In this investigation, potential chlorophyll-fluorescence-based indices were selected to detect the early heat and water stress in strawberry plants. The hyperspectral images were used to capture the fluorescence reflectance in the range of 500 nm–900 nm. From the hyperspectral cube, the region of interest (leaves) was identified, followed by the extraction of eight chlorophyll-fluorescence indices from the region of interest (leaves). These eight chlorophyll-fluorescence indices were analyzed deeply to identify the best indicators for our objective. The indices were used to develop machine-learning models to assess the performance of the indicators by accuracy assessment. The overall procedure is proposed as a new workflow for determining strawberry plants’ early heat and water stress. The proposed workflow suggests that by including all eight indices, the random-forest classifier performs well, with an accuracy of 94%. With this combination of the potential indices, namely the red-edge vegetation stress index (RVSI), chlorophyll B (Chl-b), pigment-specific simple ratio for chlorophyll B (PSSR_b_), and the red-edge chlorophyll index (CI_REDEDGE_), the gradient-boosting classifier performs well, with an accuracy of 91%. The proposed workflow works well with a limited number of training samples which is an added advantage.

## 1. Introduction

The strawberry (Fragaria × ananassa Duch) is considered one of the world’s significant fruit-yielding plants. Strawberry plants are primarily grown in the offspring season since they are vulnerable to abiotic stresses [1]. Due to impact of global climate change, these plants suffer significant heat and water stress [2]. Temperature and water stress cause changes in physiological, biological, and in-plant metabolism [3]. Its impact on strawberry plants varies according to the growing stage, based on the stress duration and growing medium. Strawberry plants have high water requirements due to their shallow root structures (with a covering area of around 0–15 cm), large leaf areas, and fruits with a high water content [4]. Moreover, there is a considerable correlation between the growth environment of horticultural crops and the quality of the plants and harvested fruits [5]. Therefore, water stress significantly reduces plant leaves’ water potential, reduces growing leaves by number, and generates disorders in photosynthesis reaction compositions [6,7]. Furthermore, heat and water stress result in a decline in fruit yield and even yield fruit sizes that are smaller than expected [8,9]. Over time, heat and water stress cause permanent damage to plants, which slows plant growth and development. Stress disorders are often diagnosed by visual symptoms and, more precisely, by mineral-exploration analysis [10]. These visible symptoms usually appear at the later stages of plant growth [11].

Identifying heat and water stress in the earlier stages is essential to enrich plant growth and production. Destructive techniques often involve mineral and protein exploration. However, the number of non-destructive techniques for detecting plant stress has increased in recent years [12], offering promising approaches for evaluating plants’ physiological and morphological properties in response to environmental changes. For example, hyperspectral imaging (HSI) is a commonly used non-destructive technique to analyze the traits of plants, which gained attention due to its contiguous scanning properties in a narrow electromagnetic spectrum. HSI provides rich spectral information and returns considerable spatial information [13]. The aforementioned properties allow researchers to utilize HSI in plant phenotyping. For instance, hyperspectral imaging was utilized for assessments of water status in different varieties of tomato plants, multi-stress environments were evaluated in lettuce plants [14], and another study reported the detection of heat-stressed ginseng plants using hyperspectral imaging systems in visible and shortwave infrared [15]. 

The photosynthesis of plants is determined by various physiological, biochemical, and plant-metabolism traits [16]. However, chlorophyll fluorescence is considered one of the major factors in understanding the photosynthesis process and its measurement is considered in identifying stress in plants [17]. The chlorophyll-fluorescence measurements extracted using HSI are non-destructive and even helpful in determining plant stress in the early stages [18]. According to previous research, fluorescence hyperspectral imaging can be employed for the detection and quantification of the health status of plants. Examples include the detection of drought stress in soybean varieties over different stress treatments [19], the early identification of head blight in winter wheat [20], and detection of scabs in apple leaves [21]. Since crops’ photosynthetic activities directly influence the physiological changes in plants, further study is necessary to comprehend the underlying mechanisms.

Chlorophyll fluorescence usually lies in the spectra’s near-infrared and red-edge regions [22].Clear studies on identifying heat and water stress at early stages have not yet been conducted. Nevertheless, these chlorophyll-fluorescence measurements can be helpful indicators when selecting stress-free plants during breeding. 

In this investigation, the heat and water stress on the strawberry plants were identified in the early stage. The HSI-fluorescence-based indices were extracted to determine the heat and water stress at the early stages. To detect the heat and water stress in the early stages, seven machine-learning models were employed, and the results were compared to obtain the best model. In this investigation, a workflow is discussed for identifying the heat and water stress on the strawberry plant in its early stages in correspondence with eight fluorescence-based indices. Specifically, this study’s main objective was to determine the potential of fluorescence hyperspectral imaging in combination with multiple machine-learning models for the early detection of heat and water stress in strawberry plants based on the extracted indices. 

## 2. Materials and Methods

The experiment was conducted in laboratory conditions. The plants were placed in a pot and kept in a controlled chamber for 7 days. The controlled chamber was set to a temperature of 25 °C for 16 h and 20 °C for 8 h. The plants were watered in the controlled chamber over 3 days for better growth. Furthermore, a nutrient solution named “Mulfuresiriz” from Daeyu Business Limited (Seoul, South Korea) was delivered hydroponically through nutrient-solution-delivery unit of plant factory with concentration of 4 mL of nutrient solution per liter of water. Secondly, the plants were transferred to the heating chamber for 6 days. In the heating chamber, the temperature was set to 35 °C for 16 h and 30 °C for 8 h. When the plants were kept in a heat chamber, no watering was conducted. Finally, the plants were transferred to the controlled chamber for 15 days for recovery. During the recovery stage, the plants were kept at a temperature of 25 °C for 16 h and 20 °C for 8 h, and watered over 3 days. The total number of plants used in the experiments was 45.

### 2.1. Imaging

The line-scan hyperspectral imaging system by Headwall Photonics (Bolton, MA, USA) was used. The primary system components consisted of a line-scan spectrograph (Headwall Photonics, Fitchburg, MA, USA) with an objective lens of (F/1.9 35 mm compact lens, Schneider Optics, Hauppauge, NY, USA). The camera was composed of an electron-multiplying charge-coupled device (EMCCD) (MegaLuca R, ANDOR Technology, South Windsor, CT, USA) with 1004 (spatial) and 1002 (spectral) pixels. The system was also equipped with two ultraviolet-illumination units (UV-365 nm, LED) (LZ4-40UA10, Ledengin Inc., Mansfield, OH, USA). The HSI system works in the visible near-infrared region (397 nm to 1003 nm) with a spectral resolution of (4.5–4.8 nm). A bandpass fluorescence filter was applied to the sensor to extract the fluorescence-based reflectance. 

**Figure 1:**
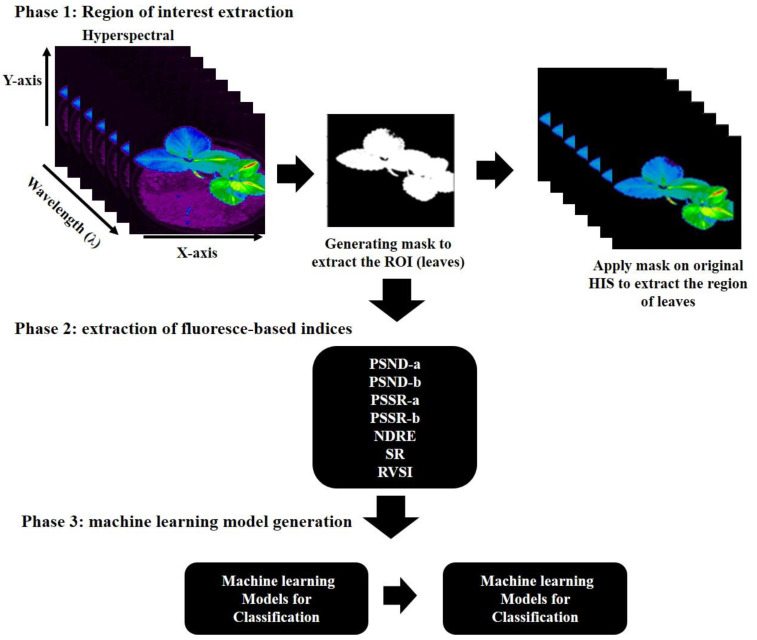
Schematic representation of data processing and model development.

In this investigation, the considered samples for imaging in the control, stressed stage, and recovery phase of the plants are listed in Table 1, and a total of 300 extracted data from the plants were divided into calibration 70% (210 samples) and validation 30% (90 samples). Furthermore, extracted data were used for processing; for better representation, a workflow was proposed consisting of three phases, from data analysis to model development, demonstrated in Figure 1. 

The proposed workflow has three phases: region-of-interest extraction, extraction of fluorescence-based indices, and machine-learning-model generation. In phase 1, a binary mask was generated using a Fast 2D Otsu Thresholding Algorithm [23]. This method is made computationally fast by avoiding redundant computations on the input HSI. Next, the obtained mask was applied to all the bands in HSI to extract the region of interest (leaves), as shown in Figure 1. In phase 2, eight chlorophyll-fluorescence-based indices were extracted from the region of interest present in the HSI. The details of the eight indices are described in a later section. In the third phase, multiple machine-learning models were employed to model plant-stress detection. The final output provided results in the form of early detection of stress, controlled, and recovered plants from the obtained chlorophyll-fluorescence-based indices.

**Figure 2:**
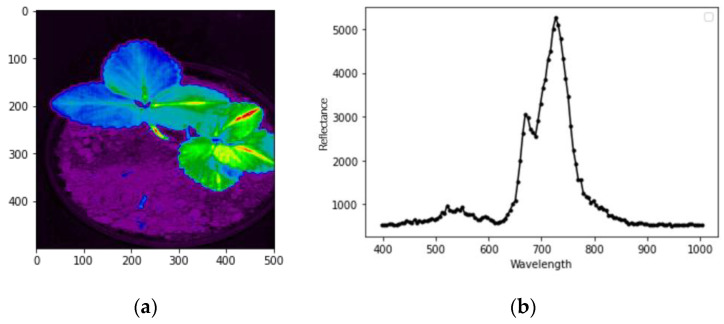
(**a**) Raw hyperspectral image; (**b**) obtained spectral means from ROI of the leaves.

Figure 2 shows the spectral graph of the image, which depicts the variation in the spectrum from 500 nm to 900 nm. Figure 2a depicts the actual HSI image retrieved using a fluorescence filter applied to the system. Figure 2b represents the spectral-reflectance curve of the obtained image in the region of interest (ROI) of the leaves. In Figure 2b, it is noticeable that the spectral-reflectance changes ranged from 500 nm to 900 nm. Chlorophyll-fluorescence metrics can usually be extracted in the near red and red edge regions (500 nm to 800 nm); this was the case in our proposed work. Details of the total data are provided in Table 1.

Table 1 A shows the number of samples that have been considered to extract spectral information from control, stressed, and recovered plants. 

### 2.2. Chlorophyll-Fluorescence Indices

In our investigation, the following eight fluorescence-based indices were used to identify stress in the early stage. These indices are representative indicators to determine chemical responses that have correlations with the concentrations of chlorophyll concentrations, which are highly correlated with near-infrared, red-edge, and visible-waveband regions [24]. 

#### 2.2.1. Pigment-Specific Normalized Difference for Chlorophyll A (PSNDa)

The correlation with the chlorophyll a significantly reduced in reflectance at R676. Since the chlorophyll does not absorb in the near-infrared region, R810 could be a possible reference value to obtain the pigment concentrations [24]. The chlorophyll absorption peak is visible at R680 in Figure 1b. Therefore, in our experiment, we took the band values closer to R676 and R810 to obtain the PSNDa. The formula to obtain the PSNDa is shown in Equation (1):

(1) $$\mathrm{PSNDa}=\frac{\mathrm{Near}\left(R_{810}\right)-\mathrm{Near}\left(R_{676}\right)}{\mathrm{Near}\left(R_{810}\right)+\mathrm{Near}\left(R_{676}\right)}$$

The values lie between −1 and +1.

#### 2.2.2. Pigment-Specific Normalized Difference for Chlorophyll B (PSNDb)

As with PSNDa, the PSNDb’s reduction in reflectance at R635 and R810 could be the possible reference value, and chlorophyll a and chlorophyll b were correlated with R635 [24]. The formula to obtain PSNDb is shown in Equation (2):

(2) $$\mathrm{PSNDb}=\frac{\mathrm{Near}\left(R_{810}\right)-\mathrm{Near}\left(R_{635}\right)}{\mathrm{Near}\left(R_{810}\right)+\mathrm{Near}\left(R_{635}\right)}$$

The values lie between −1.0 and +1.0.

#### 2.2.3. Pigment-Specific Simple Ratio for Chlorophyll A (PSSRa)

PSSR indices were well correlated with the concentrations of the chlorophylls [24]. The simple ratio for chlorophyll a is calculated as follows:

(3) $$\mathrm{PSSRa}=\frac{\mathrm{Near}\left(R_{810}\right)}{\mathrm{Near}\left(R_{676}\right)}$$

The values lie between −1.0 and +1.0.

#### 2.2.4. Pigment-Specific Simple Ratio for Chlorophyll B (PSSRb)

The simple ratio for chlorophyll b is calculated as follows:

(4) $$\mathrm{PSSRb}=\frac{\mathrm{Near}\left(R_{810}\right)}{\mathrm{Near}\left(R_{635}\right)}$$

The values lie between −1.0 and +1.0.

#### 2.2.5. Chlorophyll Index at Red Edge (CI-RedEdge)

The red-edge spectral regions were much smaller than in the near-infrared region, and in these spectral regions, light penetrated deeper into the leaf than in the near-infrared regions; therefore, absorption of light is sufficient to provide a high sensitivity of reflectance to chlorophyll contents [25]. The CI-RedEdge index was mainly dominant in red-edge areas (R700). Since the absorption was significantly less in the near-infrared region, R810 could be the possible reference value [25]:

(5) $$\mathrm{CI}-\mathrm{RedEdge}=\frac{\mathrm{Near}\left(R_{810}\right)}{\mathrm{Near}\left(R_{700}\right)}-1$$

The values lie between −1 and infinity. 

#### 2.2.6. Normalized-Difference Red-Edge Index (NDRE)

The canopy-chlorophyll index uses a normalized-difference red-edge index to detect heat and water stress [26]. The NDRE can be extracted within the reflectance range of R720–R790, as shown in Equation (6). The peak chlorophyll content at 720 nm is depicted in Figure 1b. The values lie between −1 and +1. Greater negative values indicate high stress, and lesser negative values show low stress on the plants:

(6) $$\mathrm{NDRE}=\frac{\mathrm{Near}\left(R_{790}\right)-\mathrm{Near}\left(R_{720}\right)}{\mathrm{Near}\left(R_{790}\right)+\mathrm{Near}\left(R_{720}\right)}$$

#### 2.2.7. Simple Ratio (SR)

The simple ratio defines the leaf-area index by taking the ratio of R675 and R810. The simple ratio was calculated at the defined ratio because the leaf’s red-light absorption is high at R675 and R810, where the infrared influence is very low [27]. SR calculation was carried out as shown in Equation (7). The values lie between 0 and infinity. The lesser values denote lower stress, and the greater values indicate high stress on plants:

(7) $$\mathrm{SR}=\frac{\mathrm{Near}\left(R_{810}\right)}{\mathrm{Near}\left(R_{675}\right)}$$

#### 2.2.8. Red-Edge Vegetation-Stress Index (RVSI)

The red-edge vegetation-stress index was defined as the indicator to identify the stress based on the upper-red-edge geometry [22]. The RVSI calculation was carried out using Equation (8). The values lie between −1 and +1, where the lesser values denote lower stress, and the greater values represent high stress on plants.

(8) $$\mathrm{RVSI}=\left[\frac{\mathrm{Near}\left(R_{714}\right)+\mathrm{Near}\left(R_{752}\right)\;}{2}\right]-\;\mathrm{Near}\left(R_{733}\right)$$

Only the fluorescence-based metrics were chosen for the proposed work because chlorophyll fluorescence is light re-emitted by chlorophyll molecules. This chlorophyll fluorescence can be detected by HSI, especially in range of fluorescence-related regions, and these metrics have highly potential with respect to chlorophyll-content estimation. The chlorophyll content is the critical estimator used to understand plant metabolism. Utilizing these metrics, it is possible to generate machine-learning models efficiently, with fewer samples, to identify heat and water stress at the early stage.

### 2.3. System Configuration and Packages Used

The overall analysis was carried out on a desktop with Windows 10 installed and had a RAM capacity of 32 GB. Python 3.8 was used in the backend and for reading the hyperspectral images, plantcv package was used. For analysis and extraction of metrics, pandas and NumPy were used, and for plotting the graphs, matplotlib and seaborn packages were used.

### 2.4. Machine-Learning Methods

Early detection of heat and water stress in strawberry plants improves the development and yield of all prospects. To identify these, multiple indicators were extracted, and the significant indicators were identified in the former section. This section details the multiple machine models effectively used to determine the heat and water stress in strawberry plants with all the indicators and with selective indicators. To analyze the best results, seven machine-learning algorithms were selected and categorized into three main classes. 

**Figure 3:**
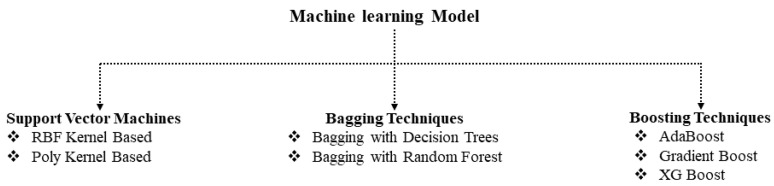
Machine-learning models used for analyzing the potential indicators.

As shown in Figure 3, seven classifiers were used to determine the significant indicators for our objective. Multiclass support vector machine (SVM) using different kernels, and two bagging techniques, namely decision-tree-based bagging and random-forest-based bagging, were used. SVM, the supervised ML method, was used to solve the binary classification and regression problem. There are four categories of kernel in SVM, including polynomial, radial basis function (RBF), sigmoid, and linear. Generally, RBF kernel-based SVM performs better than others. Bagging and boosting techniques are ensemble-machine-learning methods. Generally, bagging-ensemble machines are applied to increase classification accuracy. Bagging has proven useful in susceptibility models because of its sensitivity to slight variations in the training data [22]. Boosting ensemble machines, namely Adaboost, Gradient Boosting, and XG Boosting, are used. Boosting algorithm is broadly used and refers to a set of algorithms that transforms weak learners into strong learners [28]. Multiple classifiers were used to obtain the best model for identifying the heat and water stress on strawberry plants in accordance with the potential indicators retrieved using the HSI. The classification models were evaluated based on F1 score, precision, recall, and overall accuracy. 

## 3. Results

**Figure 4:**
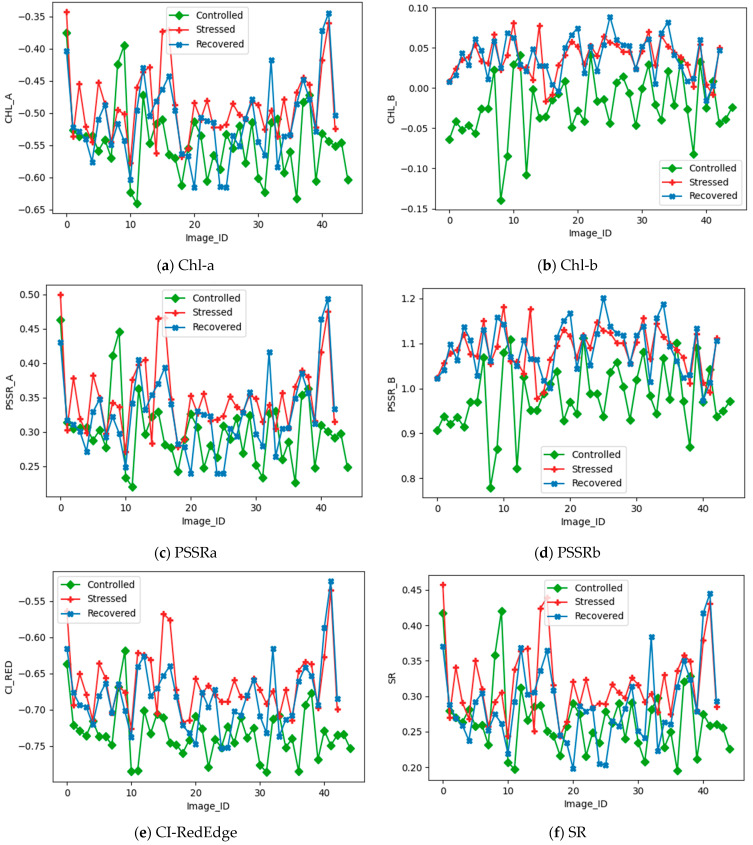

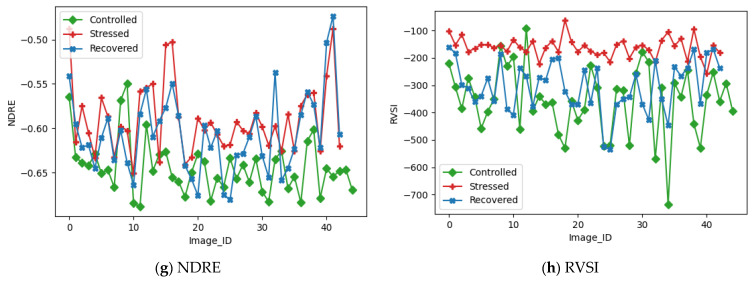
Relationships of chlorophyll-fluorescence metrics with respect to types of images (Image_ID).

The strawberry plants controlled, stressed, and recovered using the HSI were imaged, and the data are tabulated in Table 1 for reference. The region of interest was extracted to obtain the chlorophyll-fluorescence indices, and the relationship of the indices with respect to the controlled, stressed, and recovered data is depicted in Figure 4a–h. The plots in Figure 4a–h were plotted to understand the data pattern. The chlorophyll a (Chl-a) depicted in Figure 4a shows a complex non-linear pattern of data points, which is non-separable, but the chlorophyll b (Chl-b) depicted in Figure 4b shows a non-linear pattern of data points; nevertheless, the controlled one is non-linearly separable. Figure 4a,b shows that the stressed and recovered data points were difficult to separate, adding further complexity to the determination of the heat and water stress. Similarly, the PSSRa and PSSRb were plotted in Figure 4c,d, respectively. It is evident from the plots that the PSSRa exhibited a complex non-linear pattern that was non-separable, but that the PSSRb was non-linearly separable. The CI_REDEDGE_ and SR were plotted in Figure 4e,f. However, both the data patterns were non-linear complexly and non-separable, although in CI_REDEDGE_ the controlled data points were non-linearly separable. Compared to the NDRE shown in Figure 4g, the RVSI data pattern was nonlinearly separable, as shown in Figure 4h.

To understand the data distribution statistically, the correlation matrix of the controlled, stressed, and recovered data are provided in Figure 4a–c. 

**Figure 5:**
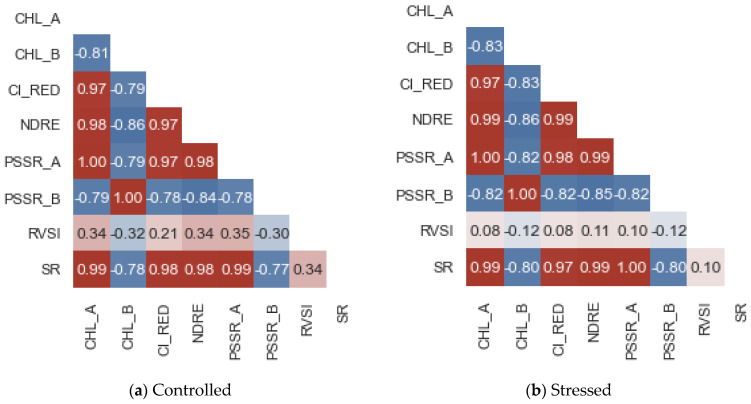

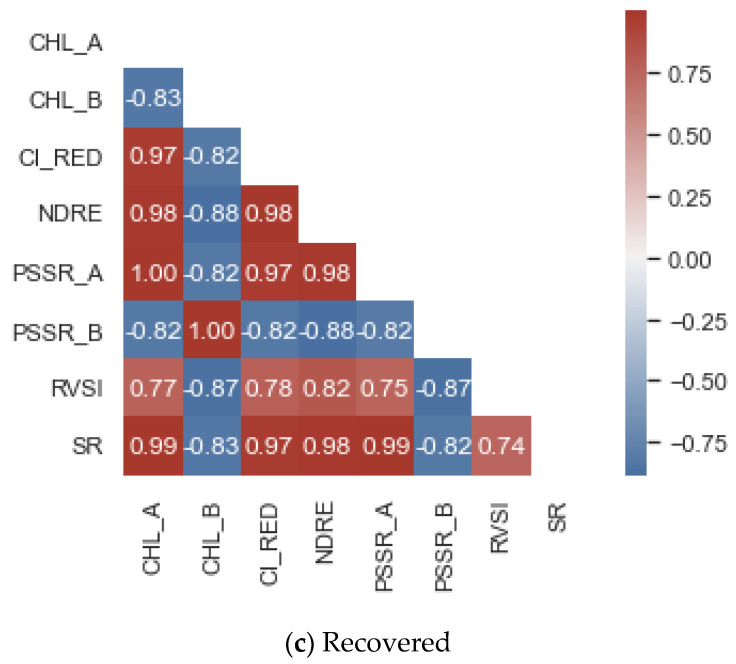
Correlation matrix of chlorophyll-fluorescence metrics at different stages. The red and blue squares represent significant positive and significant negative correlations, respectively (*p* < 0.05).

**Figure 6:**
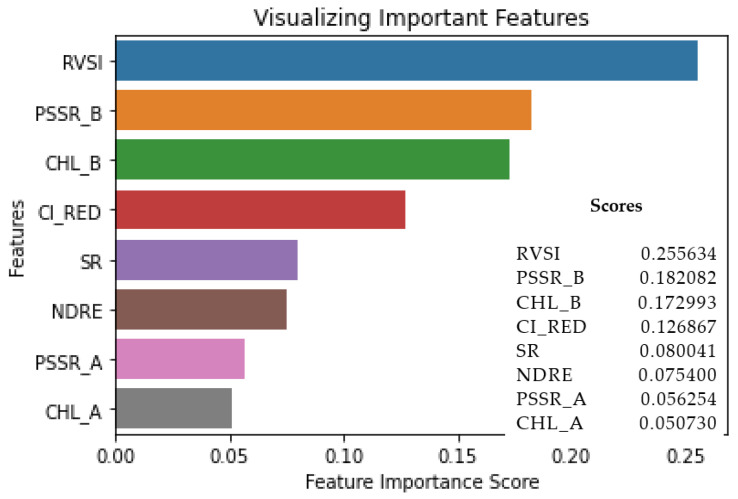
Feature-score map.

The correlation matrix shown in Figure 5 shows the best metric, which is highly correlated with other metrics at different stages. Figure 5a shows the correlation matrix of the controlled plants; it is evident from Figure 5a that the RVSI, Chl-b, and PSSRb showed better correlations against other indicators. Similar evidence can be seen in Figure 5b. However, in Figure 5c, the Chl-b and PSSRb show a better correlation against other metrics. Using Figure 3 and Figure 4, a critical observation was made to identify the best indicators among the eight chlorophyll-fluorescence indices. The indices RVSI, Chl-b, PSSRb, and CI-RedEdge offered better contributions to the identification of the heat and water stress in the early stages. To further analyze the indicators, a feature-score map was generated and depicted in Figure 6. 

As discussed above through Figure 4 and Figure 5, the feature-score map shown in Figure 6 evidently shows that the RVSI, PSSRb, Chl-b, and CI_REDEDGE_ were the most important in identifying the heat and water stress in the early stages. The indicators RVSI, PSSRb, Chl-b, and CI-RedEdge made, on statistical average, a 73% contribution to the development of the machine-learning model. However, multiple machine-learning models were analyzed with all the indicators and selective indicators to understand the best fit for our objective.

The results of the machine-learning models are tabulated with all the indicators in Table 2 and only with significant selective indicators in the Table 3. A total of 131 samples were used, including controlled, stressed, and recovered plants. In general, optimum selection variables are critical for decreasing image processing and model-development complexity. Furthermore, removing less informative data improves model performance. The feature score was calculated using standard machine-learning approaches, and a decision tree was applied through a reduction in node impurity weighted by the likelihood of the reaching node. The node-obtained probability was determined by the number of samples that reached the node divided by the total number of samples. The more significant the feature, the higher the value. The chlorophyll-fluorescence indices were extracted from the samples for analysis, as shown in Figure 4. To develop the machine-learning models, since the training samples were lower in numbers, 75% (90 samples) of the overall samples were considered for training and 25% (41 samples) for testing. The results of multiple machine-learning algorithms are tabulated with all the indices and with selective indices in Table 2 and Table 3, respectively.

## 4. Discussion

**Figure 7:**
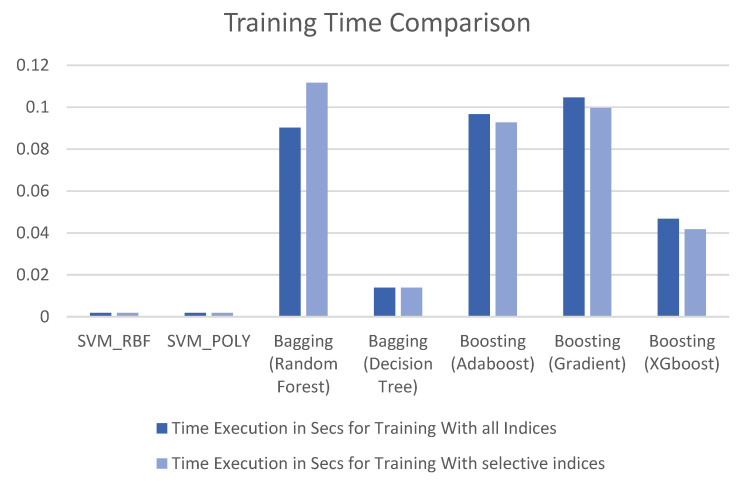
Training-time comparison of the models in second.

The best accuracy (above 80%) obtained based on the total indices is highlighted in Table 2 for a more straightforward interpretation. As shown in Table 2, when all the indices are included for training the model, the random-forest classifier yielded 94% accuracy, and the Decision Tree performed with comparably less accuracy. The results were poor with the SVM models RBF, 67%, and Polynomial, 64%, using multiple kernels because SVM usually converts the feature space into a linearly separable space. Unfortunately, in our case, the indices (features) were non-linearly separable, which is discussed in the previous section through Figure 4 and Figure 5. According to Table 2, the Boost classifier yielded an increase in accuracy of 57%, 76%, and 79% using the models AdaBoost, Gradient Boosting, and XG Boosting, respectively, which is the second-highest accuracy of all the indices. However, Table 3 shows the results of the selective indices for developing the machine-learning model. The indices were selected based on the analysis carried out in Figure 5 and Figure 6. Four potential indicators were selected, namely RVSI, Chl-b, PSSRb, and CI-RedEdge, for developing the model, and the results are tabulated in Table 3. However, Table 3 shows no improvements in SVM-based models due to the non-linearity feature pattern. Similarly, no improvements in the AdaBoost classifier were noticed. An interesting observation is that, according to Table 2 and Table 3, there was a significant improvement for bagging with the decision tree, from 70 % to 82%. On the other hand, the accuracy of the random-forest classifier declined from 94% to 85%. The XG Boosting accuracy decreased from 79% to 76% when we used selected indices. More importantly, the gradient-boosting classifier’s accuracy increased from 76% to 91% when selective indices were used. Table 2 and Table 3 evidently prove that the accuracy of multiple models improved when we used selected indices to identify the early heat and water stress on the strawberry plants. Overall, when we used all the indices, the random-forest classifier showed the best performance, and we used selective indices; the gradient boosting showed better performance in obtaining our objective. Table 2 and Table 3 clearly show that the indices RVSI, Chl-b, PSSRb, and CI-RedEdge were the best indicators for the identifying strawberry plants’ heat and water stress. The execution times of both cases are depicted in Figure 7 for illustrative purposes. When we used selective indices, the training time of the boosting techniques decreased, and on the other hand, the bagging technique’s training time increased. There was no change in SVM-based techniques.

Even though the boosting techniques’ training time decreased, the increased accuracy of the models was notable. On the other hand, the training time of the bagging-based techniques increased, but the accuracy declined with selective indices. The model training time relied on the stochasticity of each model, which might be affected by learning algorithms, the evaluation process of the variables, and differences in the platform. Specifically, it relied on the convergence time in the machine-learning algorithms; during training, different reductions in output times were observed. For example, a decision tree converged faster with more variables. The above results and discussion show that chlorophyll-fluorescence-based indices are good indicators in determining strawberry plants’ early heat and water stress. Interestingly, the proposed workflow offers the added advantage of a smaller number of samples. Therefore, the indicators used in the proposed work best determine strawberry plants’ heat and water stress.

## 5. Conclusions

In this investigation, we identified potential indicators for identifying the early heat and water stress on strawberry plants. The proposed workflow is a robust approach to achieving our objective at any stage. We performed a deeper analysis of all the indicators and selective indicators to detect heat and water stress. The analysis proved that the indicators’ non-linearity at different stages made the system model complex. Multiple machine-learning algorithms were used to evaluate the indicator performance through accuracy assessment. As a concluding remark, random forest used all the indicators to return the best accuracy. To obtain the best accuracy, the gradient-boosting algorithm used only selective indicators, namely RVSI, Chl-b, PSSRb, and CI-RedEdge. This investigation proves that the chlorophyll-fluorescence indicators extracted from HSI are the most suitable for identifying heat and water stress in strawberry plants. The proposed workflow is scalable in canopy-level heat-and-water-stress detection in strawberry fields, which is an added advantage.

## Figures and Tables

**Table 1:** Details of the data.

Type	Count	Resolution
Controlled	45	502 × 500 × 128
stressed	43	502 × 500 × 128
Recovered	43	502 × 500 × 128

**Table 2:** Results of machine-learning models including all the indicators.

Models and Scores	Class	SVM		Bagging		Boosting		
		RBF	Polynomial	Decision Tree	Random Forest	Adaboost	Gradient Boosting	XG Boosting
Precision	Controlled	0.67	1.00	1.00	1.00	1.00	0.86	0.88
	Stressed	0.56	0.50	0.44	0.80	0.35	0.58	0.64
	Recovered	0.73	0.71	0.62	1.00	0.00	0.86	0.86
Recall	Controlled	0.46	0.15	0.85	0.92	0.92	0.46	0.54
	Stressed	0.62	0.88	0.50	1.00	0.88	0.88	0.88
	Recovered	0.92	1.00	0.67	0.92	0.00	1.00	1.00
F1 Score	Controlled	0.55	0.27	0.92	0.96	0.96	0.60	0.67
	Stressed	0.59	0.64	0.47	0.89	0.50	0.70	0.74
	Recovered	0.81	0.83	0.64	0.96	0.00	0.92	0.92
Overall Accuracy		0.67	0.64	0.70	0.94	0.57	0.76	0.79

**Table 3:** Results of machine-learning models including selective indicators (RVSI, Chl-b, PSSR_b_, and CI_REDEDGE_).

Models and Scores	Class	SVM		Bagging		Boosting		
		RBF	Polynomial	Decision Tree	Random Forest	Adaboost	Gradient Boosting	XG Boosting
Precision	Controlled	0.67	1.00	1.00	0.91	1.00	0.92	0.86
	Stressed	0.56	0.50	0.73	0.78	0.35	0.78	0.58
	Recovered	0.73	0.71	0.79	0.85	0.00	1.00	0.86
Recall	Controlled	0.46	0.15	0.62	0.77	0.92	0.85	0.46
	Stressed	0.62	0.88	1.00	0.88	0.88	0.88	0.88
	Recovered	0.92	1.00	0.92	0.92	0.00	1.00	1.00
F1 Score	Controlled	0.55	0.27	0.76	0.83	0.96	0.88	0.60
	Stressed	0.59	0.64	0.84	0.82	0.50	0.82	0.70
	Recovered	0.81	0.83	0.85	0.88	0.00	1.00	0.92
Overall Accuracy		0.67	0.64	0.82	0.85	0.57	0.91	0.76

## Data Availability

Data sharing is not applicable to this article.
